# *Notes from the Field*: Overdose Deaths Involving *Para*-fluorofentanyl — United States, July 2020–June 2021

**DOI:** 10.15585/mmwr.mm7139a3

**Published:** 2022-09-30

**Authors:** Jessica Bitting, Julie O’Donnell, Christine L. Mattson

**Affiliations:** ^1^Division of Overdose Prevention, National Center for Injury Prevention and Control, CDC; ^2^National Network of Public Health Institutes, Washington, DC.

Provisional estimates indicate that synthetic opioids, including illicitly manufactured fentanyl (IMF), were involved in approximately two thirds of an estimated 108,174 overdose deaths in the United States during the 12 months ending in April 2022.* Previous analyses have identified *para*-fluorofentanyl, a schedule I^†^ illicit fentanyl analog, in drug overdose deaths in eight states from late 2020 through June 2021 (*1*–3). Limited data suggest that *para*-fluorofentanyl is likely similar to or slightly less potent than IMF (*3**,**4*); however, its role in the illicit drug market and its impact on the opioid overdose crisis has not been widely studied. To better understand monthly trends in drug overdose deaths involving *para*-fluorofentanyl in the United States, CDC analyzed overdose death data from the State Unintentional Drug Overdose Reporting System (SUDORS).

SUDORS includes data from death certificates and medical examiner and coroner reports (including enhanced postmortem toxicology testing) on unintentional and undetermined-intent drug overdose deaths. CDC assessed monthly frequencies of overdose deaths during July 2020–June 2021 involving (i.e., listed as a cause of death) *para*-fluorofentanyl, among 42 states^§^ and the District of Columbia. *Para*-fluorofentanyl–involved deaths were stratified by jurisdiction and U.S. Census Bureau region.^¶^ This activity was reviewed by CDC and conducted consistent with applicable federal law and CDC policy.**

*Para*-fluorofentanyl was involved in 1,658 (2.6%) of 64,915 overdose deaths reported by 43 jurisdictions during July 2020–June 2021. *Para*-fluorofentanyl–involved deaths increased from the first reported occurrences in September 2020 (five deaths) through a peak of 293 deaths in May 2021 (Figure). The number of *para*-fluorofentanyl–involved deaths increased 455.3% from 253 during July–December 2020 to 1,405 during January–June 2021. Deaths involving *para*-fluorofentanyl occurred in 35 jurisdictions and accounted for 3.9%, 2.9%, 1.9%, and 1.1% of overdose deaths in included jurisdictions in the Northeast, South, Midwest, and West U.S. Census Bureau regions, respectively. Six states (Illinois, Maryland, Michigan, New Jersey, Pennsylvania, and Tennessee) reported more than 100 deaths involving *para*-fluorofentanyl. *Para*-fluorofentanyl–involved deaths nearly always co-involved IMF^††^; co-involvement ranged from 100% of deaths in September 2020 to 90.8% in June 2021.

**FIGURE:**
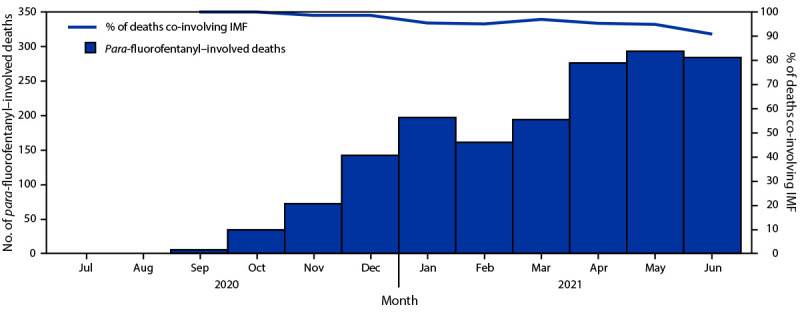
Number of *para-*fluorofentanyl–involved drug overdose deaths and percentage co-involved with illicitly manufactured fentanyl* — State Unintentional Drug Overdose Reporting System, United States, July 2020–June 2021 **Abbreviation:** IMF = illicitly manufactured fentanyl. * IMF excludes fentanyl analogs.

The findings in this report are subject to at least three limitations. First, analyses were limited to 43 jurisdictions and might not be generalizable to the entire United States. Second, although comprehensive postmortem testing protocols recommend IMF testing (*5*), lack of standard testing requirements might lead to an underestimation of *para*-fluorofentanyl involvement in drug overdose deaths. The rise in *para*-flourofentanyl detection could also be caused by increases in testing during the study period. Finally, death certification training and experience vary across and within medical examiner and coroner systems, potentially leading to differences in *para*-fluorofentanyl’s inclusion as the cause of death even when it is detected.

The emergence of *para-*fluorofentanyl involvement in deaths in 35 SUDORS-funded jurisdictions supports and furthers evidence of recent increases (*1*–*3*). Because of high co-involvement with IMF, it is unclear whether the proliferation of *para*-fluorofentanyl reflects a diversification of the illicit drug market (i.e., *para*-fluorofentanyl is being mixed with IMF) or it has emerged as a new stand-alone product. Because data on potency are limited, it is unclear whether *para*-fluorofentanyl poses a higher risk than does fentanyl alone; however, access to and timely administration of naloxone to reverse opioid overdoses (*1*), as well as ensuring access to substance use prevention and treatment services, including distribution of fentanyl test strips, is crucial to prevent *para*-fluorofentanyl overdose deaths. In addition, because the illicit drug market continues to evolve rapidly and some jurisdictions might have a lack of or limited testing capabilities, a critical need exists for expanded, enhanced toxicology testing to detect *para*-fluorofentanyl and other emerging drugs.

## Footnotes

*
https://www.cdc.gov/nchs/nvss/vsrr/drug-overdose-data.htm

† Schedule I drugs, substances, or chemicals are defined as drugs with no currently accepted medical use and a high potential for abuse. https://www.dea.gov/drug-information/drug-scheduling

§ Jurisdictions included Alabama, Alaska, Arizona, Arkansas, Colorado, Connecticut, Delaware, District of Columbia, Georgia, Hawaii, Illinois, Indiana, Iowa, Kansas, Kentucky, Louisiana, Maine, Maryland, Massachusetts, Michigan, Minnesota, Mississippi, Missouri, Montana, Nebraska, Nevada, New Hampshire, New Jersey, New Mexico, North Carolina, Ohio, Oklahoma, Oregon, Pennsylvania, Rhode Island, South Carolina, South Dakota, Tennessee, Utah, Vermont, Virginia, Washington, and West Virginia. Alabama, Illinois, Indiana, Louisiana, Missouri, Pennsylvania, and Washington reported deaths from counties that accounted for ≥75% of drug overdose deaths in the state in 2017, per SUDORS funding requirements; all other jurisdictions reported deaths from the full jurisdiction.

¶ Not all jurisdictions in each region are included in analyses; trend analyses include eight of nine jurisdictions in the Northeast Region (Connecticut, Maine, Massachusetts, New Hampshire, New Jersey, Pennsylvania, Rhode Island, and Vermont); 10 of 12 jurisdictions in the Midwest Region (Illinois, Indiana, Iowa, Kansas, Michigan, Minnesota, Missouri, Nebraska, Ohio, and South Dakota); 15 of 17 jurisdictions in the South Region (Alabama, Arkansas, Delaware, District of Columbia, Georgia, Kentucky, Louisiana, Maryland, Mississippi, North Carolina, Oklahoma, Tennessee, South Carolina, Virginia, and West Virginia); and 10 of 13 jurisdictions in the West Region (Alaska, Arizona, Colorado, Hawaii, Montana, Nevada, New Mexico, Oregon, Utah, and Washington). https://www2.census.gov/geo/pdfs/maps-data/maps/reference/us_regdiv.pdf

** 45 C.F.R. part 46.102(l)(2), 21 C.F.R. part 56; 42 U.S.C. Sect. 241(d); 5 U.S.C. Sect. 552a; 44 U.S.C. Sect. 3501 et seq.

†† Fentanyl was classified as likely illicitly manufactured using toxicology, scene, and witness evidence. In the absence of sufficient evidence to classify fentanyl as illicit or prescription (fewer than 12% of deaths involving fentanyl), fentanyl was classified as illicit because most fentanyl overdose deaths involve IMF. All fentanyl analogs were excluded.
